# An analysis of training load in highly trained female football players

**DOI:** 10.1371/journal.pone.0299851

**Published:** 2024-03-28

**Authors:** Andreas K. Winther, Ivan Baptista, Sigurd Pedersen, João Brito, Morten B. Randers, Dag Johansen, Svein Arne Pettersen

**Affiliations:** 1 Faculty of Health Sciences, School of Sport Sciences, UiT the Arctic University of Norway, Tromsø, Norway; 2 Faculty of Science and Technology, Department of Computer Science, UiT the Arctic University of Norway, Tromsø, Norway; 3 Faculty of Sport, Center for Research, Training, Innovation and Intervention in Sport (CIFI2D), University of Porto, Porto, Portugal; 4 Portugal Football School, Portuguese Football Federation, Lisbon, Portugal; 5 Faculty of Health Sciences, Department of Sports Science and Clinical Biomechanics, SDU Sport and Health Sciences Cluster, University of Southern Denmark, Odense, Denmark; Portugal Football School, Portuguese Football Federation, PORTUGAL

## Abstract

This observational study aimed to analyze external training load in highly trained female football players, comparing starters and non-starters across various cycle lengths and training days. **Method:** External training load [duration, total distance [TD], high-speed running distance [HSRD], sprint distance [SpD], and acceleration- and deceleration distance [AccDec_dist_] from 100 female football players (22.3 ± 3.7 years of age) in the Norwegian premier division were collected over two seasons using STATSports APEX. This resulted in a final dataset totaling 10498 observations after multiple imputation of missing data. Microcycle length was categorized based on the number of days between matches (2 to 7 days apart), while training days were categorized relative to match day (MD, MD+1, MD+2, MD-5, MD-4, MD-3, MD-2, MD-1). Linear mixed modeling was used to assess differences between days, and starters vs. non-starters. **Results:** In longer cycle lengths (5–7 days between matches), the middle of the week (usually MD-4 or MD-3) consistently exhibited the highest external training load (~21–79% of MD TD, MD HSRD, MD SpD, and MD AccDec_dist_); though, with the exception of duration (~108–120% of MD duration), it remained lower than MD. External training load was lowest on MD+2 and MD-1 (~1–37% of MD TD, MD HSRD, MD SpD, MD AccDec_dist_, and ~73–88% of MD peak speed). Non-starters displayed higher loads (~137–400% of starter TD, HSRD, SpD, AccDec_dist_) on MD+2 in cycles with 3 to 7 days between matches, with non-significant differences (~76–116%) on other training days. **Conclusion:** Loading patterns resemble a pyramid or skewed pyramid during longer cycle lengths (5–7 days), with higher training loads towards the middle compared to the start and the end of the cycle. Non-starters displayed slightly higher loads on MD+2, with no significant load differentiation from MD-5 onwards.

## Introduction

In high-performance sports, a key challenge for coaches and players is striking the right balance between training and recovery. On the one hand, increases in duration, frequency, and intensity of training are often associated with an enhancement in performance [1–3]. On the other hand, increases in training load without adequate recovery may hinder performance and increase the potential risk of injury [4]. In order to find this balance, the periodization of training is considered to be a critical tool [5]. This involves sequencing the overall training plan into units of different lengths (i.e., macro- and microcycles) and planning specific training activities and intensities for each unit [6,7]. Through careful planning and monitoring, players are believed to maintain a healthy balance between pushing their physical limits and allowing for adequate recovery [5].

In football, variations in training load are most frequently seen at the microcycle level [8]. This is because microcycles can easily be manipulated based on the number of days between matches, allowing practitioners to plan loads that provide a physical stimulus to the players and facilitate recovery [8,9]. More recently, a principle known as “horizontal alternation” [10] has often been mentioned in tandem. This principle encompasses the idea that physical capacities such as strength, endurance, or speed are targeted on specific days, potentially maximizing the stimuli of each capacity while at the same time minimizing any physiological interferences [11]. This is often done within “days before the match” (MD-) and/or “days after a match” (MD+) framework. To give an example, with six days between matches, three “acquisition” days (MD-4, MD-3, and MD-2) could be placed in between one or two “recovery” days (MD+1 to MD+2) and one “tapering” day (MD-1), where then each “acquisition” day could be dedicated to a specific capacity. In this way, all capacities are maintained or further developed while allowing players enough time to recover between matches.

In professional football, the widespread adoption of Global Positioning System (GPS)-based tracking systems has become prevalent for monitoring the players’ activity profiles. These systems can provide practitioners with numerous metrics about a player’s external training load and are considered valid and reliable in this respect [12]. Regarding the metrics themselves, both total distance and metrics describing distances covered at various speeds and accelerations and decelerations are typical metrics that both coaches and players want to see [13,14]. This extends to the planning and monitoring of training, where said metrics could be used as indicators for whether a physical capacity was appropriately targeted. For example, one could expect a more “strength” oriented day to coincide with more accelerations and decelerations [15] due to smaller pitch sizes allowing for more duals and changes of direction. In the same manner, a more endurance-focused day could coincide with longer training durations and total distance covered and a “speed” day with more distance covered at higher speeds [16].

To date, only a few studies have analyzed the periodization of training load in women’s football. Most recently, Karlsson et al. [15] found that a Norwegian team differentiated their training load in longer cycles (with 5–7 training days available), closely resembling the horizontal alternation principle. In cycles with four days between matches, Diaz-Seradilla et al. [17] found that MD was more demanding than any training day, while all external training load variables were higher on MD-3 compared to any other training day. Romero-Moraleda et al. [18] also found that the match was the most demanding session in cycles with five days between matches while observing that the training load followed a pyramid shape in which the MD-4 and MD-3 consistently produced the greatest physiological and biomechanical loads, and MD+1 the lowest values.

While research has examined differences between training days in some cycle lengths, little is known about the training load across a broad range of cycles. Furthermore, all previous studies have only investigated players with over 60 minutes of playing time, meaning little is known about the training load of non-starters. Thus, this study aimed to analyze the external training load across a range of typical cycle lengths in professional football, including potential differences between starters and non-starters. We hypothesized that teams differentiated their training load, especially during longer cycles, and that non-starters had higher training loads on MD+1 and MD+2.

## Methods

Before commencing the study, we applied for ethical approval through the Regional Committee for Medical and Health Research Ethics—Northern Norway (reference number 53884). We were exempted since the data collection did not include a biobank, medical or health data related to illness, or interfered with the regular operation of the players. After approval from the Norwegian Centre for Research Data (reference number: 296155), we obtained written informed consent from 100 female football players (22.3 ± 3.7 years of age) representing four teams in the Norwegian premier division, classified as highly trained according to the criteria outlined by McKay et al. [19]. Starting in March 2020, a prospective observational study was conducted in which tracking data from training and matches over two full seasons were collected using STATSports Apex (Newry, Northern Ireland), with a sampling frequency of 10 Hz. The validity and level of accuracy (bias <5%) of this tracking system have been previously presented [20]. All teams trained and played home matches on artificial grass, with only occasional away games on natural grass. Training sessions were usually started between 10 AM and 4 PM, with matches typically played between 1 PM and 9 PM. During training and matches, players wore their GPS unit on their upper back, adhering to manufacturer instructions. Furthermore, to minimize inter-device errors [20], each player used the same GPS unit throughout data collection. For the study, we only included outfield players and players with at least one appearance in an official match lineup, either as a starter or as a bench player.

### Data pre-processing

Following GPS reporting standards [21], we exported raw GPS data from the manufacturer’s software (STATSports Sonra 2.1.4, Newry, Northern Ireland) into a Python (3.9.12) script for pre-processing. Here, we applied a 1-second moving average to smooth doppler-derived speed and derive distance and acceleration. Next, another custom script calculated the physical performance variables. These included duration (measured using timestamps from the raw data), peak speed, total distance (TD), high-speed running distance (HSRD) (>16 km∙h^-1^), and sprint distance (SpD) (>20 km∙h^-1^) based on previous research [22–24]. In addition, combined acceleration- and deceleration distance (AccDec_dist_) was defined as the distance covered with a positive or negative change in speed of more than ± 2.26 m∙s^-2^, finishing when the rate of acceleration/deceleration reached 0 m∙s^-2^.

After deriving all the metrics, the data were transferred to an R 4.0.5 [25] script for missing data imputation and statistical analysis. All variables included in the final analysis are listed in Table 1.

**Table 1:** Overview of variables included in the final analysis.

Variable	Threshold	Type	Units
Duration		Continuous	Minutes (min)
TD		Continuous	Meters (m)
HSRD	>16 km•h^-1^	Continuous	m
SpD	>20 km•h^-1^	Continuous	m
AccDec_dist_,	>2.26 m•s^-2^	Continuous	m
Peak speed		Continuous	Meters per seconds (m∙s^-1^)
Match day and cycle		Nominal	MD, MD+2x3, MD-1x3, MD+2x5, MD-3x5, MD-2x5, MD-1x5, MD+2x6, etc.
Squad status		Nominal	Starter, non-starter
Player ID		Nominal	
Team ID		Nominal	

TD–Total distance; HSRD–high-speed running distance; SpD–Sprint distance; AccDec_dist_−Acceleration and Deceleration distances; MD–Match-day.

### Handling of missing data

To handle missing data, we followed recommendations by Bache-Mathiesen et al. [26], Borg et al. [27], and Malone et al. [21]. First, we set all physical performance variabbles as missing on sessions with a mean horizontal dilution of precision >5 or a mean number of satellites <12. We also set peak speed as missing if above 32 km∙h^-1^ based on theoretical max speed values of 29.2 ± 1.4 km∙h^-1^ in a similar cohort [28].

The initial dataset included one observation for each squad player for each day throughout the competitive season (lasting 157 and 176 days in 2020 and 2021, respectively), totaling 12879 observations, with 7646 missing. We opted to remove all observations on MD+1 since it typically was a day off with a substantial amount of missing data (2208 out of 2426 observations). We also removed all observations in cycles with four training days due to too few observations (171 in total with 132 missing). An overview of missing values in the final dataset is shown in Table 2.

**Table 2:** Number of missing and non-missing observations.

MD (+-)	Cycle	Total non-missing	Total missing	# of players	Mean # of non-missing obs. p/player	Mean HDOP	Mean # of satellites
MD		1158	527	100	11.9	1.3	18.7
MD + 2	2	181	106	95	3.5	2.0	19.8
MD + 2	3	228	413	100	2.9	1.5	19.6
MD—1		392	247	100	4.8	1.7	19.6
MD + 2	5	133	415	100	1.8	1.7	18.5
MD—3		333	215	100	3.4	1.6	19.1
MD—2		208	340	100	2.7	1.6	18.9
MD—1		314	234	100	3.3	1.8	19.4
MD + 2	6	210	391	99	2.7	1.5	18.7
MD—4		366	235	99	3.9	1.5	18.5
MD—3		346	254	99	3.8	1.5	18.6
MD—2		145	455	99	3.5	1.1	17.7
MD—1		280	320	99	3.7	1.6	19.5
MD + 2	7	69	243	100	1.6	1.4	17.6
MD—5		174	138	100	2.0	1.6	18.7
MD—4		127	185	100	1.7	1.5	18.9
MD—3		103	209	100	1.7	1.7	19.8
MD—2		56	256	100	1.1	2.0	18.9
MD—1		153	159	100	2.1	1.5	19.8

MD–match-day; #—number; HDOP–horizontal dilution of precision; p/player–per player.

We used multiple imputation with predicted mean matching (PMM) to impute the missing data, consistent with Bache-Mathiesen et al. [26]. Using the mice package [29] in R, we applied the PMM (mice.impute.pmm) method, including all dependent variables in addition to day number, to generate five imputed datasets for subsequent analysis.

### Statistical analysis

Duration, TD, peak speed, and AccDec_dist_ were modelled in R using the lmer package [30], while HSRD and SpD were modelled in the same software using glmmTMB [31]. All models included the interaction between match day, cycle, and squad status as fixed effects and player ID and team ID as random effects. In addition, HSRD and SpD were modelled using the tweedie family with a log link function. Next, we examined, only for the starters, the differences in training load between each day within each cycle and then compared the differences in training load between starters and non-starters within each day. Here, the package emmeans [32] was used to compute estimated marginal means, using the Sidak method to adjust for multiple comparisons between the days and the Tukey method for pairwise comparison between starter and non-starters. We also conducted the same statistical analysis on the non-imputed dataset with only complete cases for sensitivity purposes. Unless otherwise stated, all results are reported as estimated marginal means ± 95% confidence intervals.

## Results

**Fig 1:**
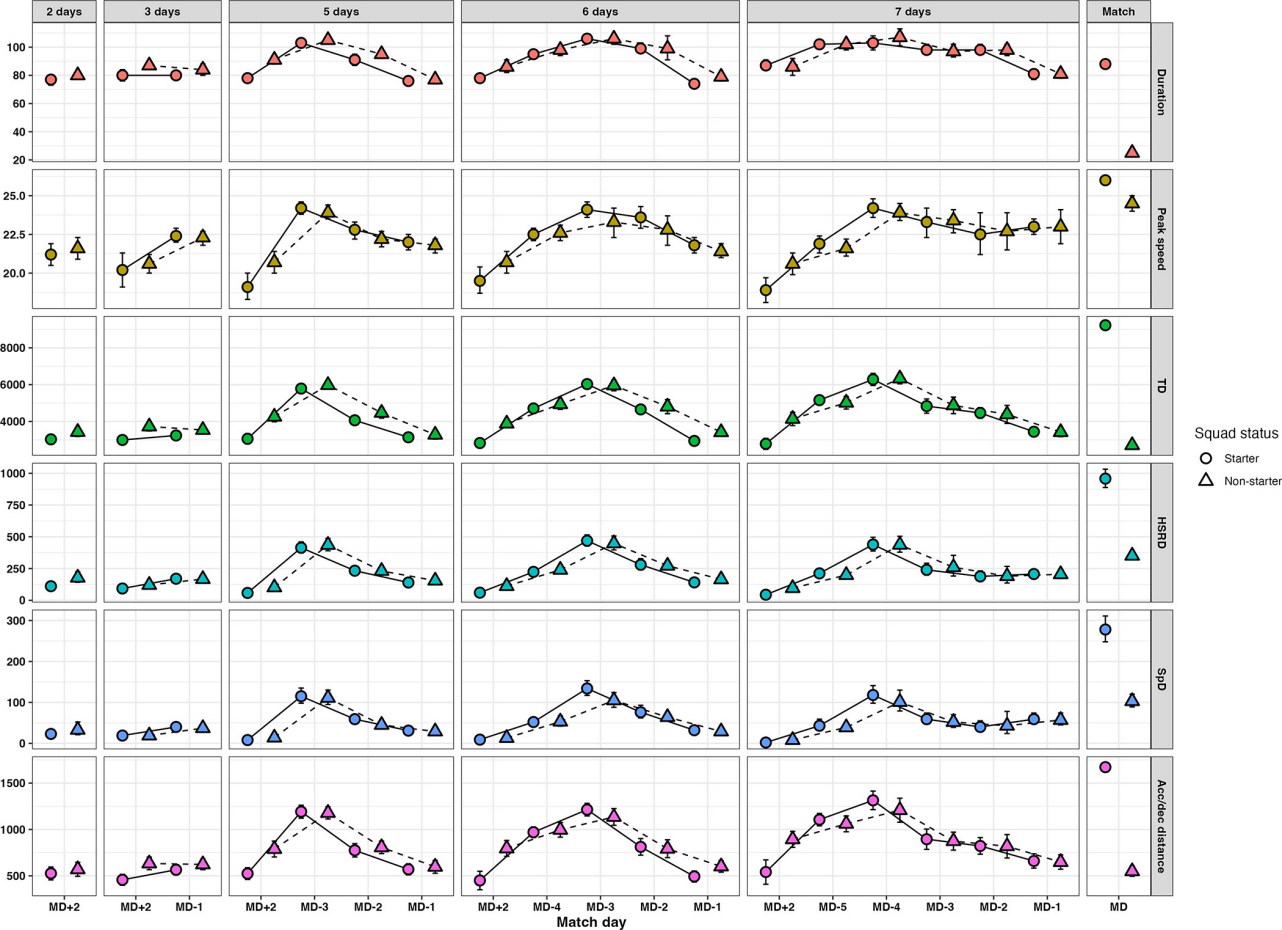
External training load by number of days between matches and in proximity to match day (imputed data).

Results from the imputed datasets and subsequent models are shown in S1–S3 Tables and Fig 1. Overall, both multiple imputation and complete case analysis gave similar results, and thus only the multiple imputation results are described below. The results for the complete case analysis can be found in S4–S6 Tables and S1 Fig.

### Match vs. training

Starters displayed significantly higher values (*p* < 0.001) for TD, HSRD, SpD, AccDec_dist_ and peak speed on MD compared to any other day. MD duration was approximately 88 ± 1 min, shorter (7 ± 4 to 18 ± 4 min, *p* < 0.001) than training on most acquisition days (MD-5 to -3) in cycles with 5–7 days between matches.

### Three days between matches

With three days available (1280 observations), there were no significant differences in duration and TD between MD+2 and MD-1. However, AccDec_dist_, HSRD, SpD and peak speed were slightly higher on MD-1 compared to MD+2, with differences of 108 ±91(*p* = 0.005), 77 ± 38 m (*p* < 0.001), 21 ±13 (*p* < 0.001), and 2.2 ± 1.2 km∙h^-1^ (*p* = 0.01), respectively.

### Five days between matches

In cycles with five days between matches (2192 observations), TD, HSRD, SpD, AccDec_dist_ and peak speed were all lower on MD+2 compared to the other training days, except for TD (81 ± 493 m, *p* = 1.000) and AccDec_dist_ (47 ± 115 m, *p* = 1.000) on MD-1. Differences in TD and mean peak speed ranged from 2728 ± 434 to 1005 ± 597 m, and from 2.9 ± 1.9 to 5.0 ± 1.5 km∙h^-1^, respectively, whilst differences in HSRD and SpD ranged from 82 ± 49 to 356 ± 74 m and from 24 ± 13 to 108 ± 30 m. Differences in AccDec_dist_ ranged from 668 ±122τo 251 ± 121 m. All variables were higher (*p* < 0.001) on MD-3 compared to the other days of the cycle, with the largest differences observed when compared to MD+2 and MD-1, respectively.

### Six days between matches

In six-day cycles (3002 observations), all variables were higher on MD-4 to MD-2 compared to MD+2 (*p* < 0.001). Similarly, both TD (ranging from 1712 ± 430 to 3087 ± 380 m), HSRD (82 ± 42 to 353 ± 111 m), SpD (20 ± 15 to 102 ± 27 m), and AccDec_dist_ (320 ± 137 to 721 ± 110 m) were higher on MD-4 to MD-2 compared to MD-1. However, statistically non-significant differences in peak speed (0.7 ± 0.8 km∙h^-1^, *p* = 0.158) were found between MD-4 and MD-1. Furthermore, MD-3 had higher duration (11 ± 5 min, *p* < 0.001) and higher peak speeds (1.5 ± 1.0 km∙h^-1^, *p* < 0.001) compared to MD-4, and higher TD (1323 ± 370 and 1374 ± 463 m, *p* ≤ 0.001), HSRD (245 ± 65 and 188 ± 84 m, *p* < 0.001), SpD (81 ± 26 and 58 ± 29 m, *p* < 0.001) and AccDec_dist_ (244 ± 103 and 408 ± 182 m, *p* < 0.001) compared to both MD-4 and MD-2. The only difference between MD-4 and MD-2 was in AccDec_dist_ (157 ± 156 m, *p* = 0.047) and peak speed (1.1 ± 1.1 km∙h^-1^, *p* = 0.047), with higher AccDec_dist_ covered on MD-4, and higher peak speed on MD-2.

### Seven days between matches

Seven-day cycles (1872 observations) saw a similar pattern to that of five and six, with all variables being higher on MD-5 to MD-3 compared to MD+2. There were also differences in the tapering stage of the cycle, with longer (11 ± 10 min, *p* ≤ 0.015) practice time on MD-2 compared to MD-1, coupled with more TD (1021 ± 602 m, *p* < 0.001) and AccDec_dist_ (162 ± 142 m, *p* = 0.009) covered. TD, HSRD, SpD and AccDec_dist_ were higher on MD-4 than any other training day. AccDec_dist_ was higher MD-5 versus MD-3 (211 ± 171 m, *p* = 0.004).

### Starters vs non-starters

Starters vs. non-starters displayed mostly small and non-significant differences in external training load, except on MD+2. Non-starters trained longer (7 ± 5 to 13 ± 4 min, *p* ≤ 0.001) in cycles with 3–6 days between matches, resulting in more TD (731 ± 246 to 1197 ± 218 m, *p* < 0.001), AccDec_dist_ (176 ± 68 to 346 ± 106 m, *p* < 0.001), HSRD (28 ± 23 to 51 ± 26 m, *p* ≤ 0.019) and higher peak speeds (1.2 ± 1.2 to 1.7 ± 0.7 km∙h^-1^) on those days.

## Discussion

Our study aimed to analyze the external training load across a range of typical cycle lengths in professional football, including potential differences between starters and non-starters. We hypothesized that teams differentiated their training load, especially during longer cycles, and that non-starters had higher training loads on MD+1 and MD+2. In line with this, two major findings were apparent from this study. First, the results indicate that the teams in our study altered their external load based on the number of days between matches, with most of the training load clustered towards the mid-week, succeeding and preceding days of lower loads. Secondly, there was little to no differentiation in training load between starters and non-starters from MD-5 and onwards, regardless of cycle.

Our data indicates that teams perform the highest combined external load at least three to four days pre-match in a typical match fixture. This period of higher load succeeds and precedes days of lower load, which makes sense from a periodization standpoint. This forms a basic structure where the first few days post-match are usually geared towards recovery, mid-week towards acquisition, and pre-match towards tapering. However, in shorter cycles with only two or three days between matches, our data indicates that most of the time is spent at lower loads awaiting a mid-week game. Regardless of metric, however, the loads are lower than match day, though there is less difference for AccDec_dist_ than for SpD. For example, AccDec_dist_ on MD-3 and MD-4 in longer cycles (5–7 days between matches) is 59–79% of MD, while SpD is only 22–51%. This could be due to a preference for small-sided games, which often involve a smaller area, hence giving insufficient space to accumulate distances at higher speeds [33].

Regarding the training day differentiation, we found no apparent differences in SpD between MD-4 and MD-2 and between MD-5 and MD-3 in cycles of six and seven days between matches. However, for AccDec_dist_, we did find significantly more distance covered on MD-4 and MD-5 versus MD-2 and MD-3, which could suggest a day with smaller spaces, while there was a tendency for higher peak speeds on MD-2 and MD-3. It is also interesting to note that the highest estimated mean peak speed in training was 24.2 km∙h^-1^ or ~93% of the estimated mean peak speed on MD for starters (26.0 km∙h^-1^). Considering that Haugen et al. [28] found theoretical peak speed values of 29.2 ± 1.4 km∙h^-1^, or roughly ~112% of match day, we are looking at a difference of ~19% between what players could be physically able to achieve versus what they are achieving in training. Added to the fact that training load decreases throughout the season [15,34], this could explain why we also see a concurrent decrease in sprint ability during this period [34]. From a specificity standpoint, coaches should be aware of the importance of training at maximum running speed to enhance this capacity [35]. In addition, being exposed to maximum speeds could also be important from an injury prevention standpoint, as exposure to high-speed football actions has been suggested to be a modifiable risk factor for hamstring injuries [36,37].

Continuing with the second finding, it is more challenging to discern whether load compensation is given for the substitutes in the combination of cycles and days available. Although there were some differences between starters and substitutes in training duration, TD, peak speed, and AccDec_dist_ on MD+2 in most cycles, this could be due to residual fatigue from the last match in the starters. There were also no pronounced differences in HSRD and SpD, and the overall load was considerably than any other day. However, this does not exclude the fact that substitute compensation could have occurred at MD or MD+1 or both. For example, training could be executed in forms that do not require tracking equipment since there were huge amounts of missing data at MD+1. Of note is that the teams in our sample had both a second team and a junior team, and it is likely that match play at these competitive levels was given as compensation, again, without it being tracked.

Our results are comparable to other studies on female football players, most notably to Karlsson et al. [15], which also included a team from the Norwegian premier division. Overall, the loading patterns and distances covered were fairly similar, with MD-4 and MD-3 dependent on cycle, equivalent to MD-3 in Karlsson et al. [15], being the training day with the highest external load for most metrics. That external training load is higher on MD-3 in cycles with five days between matches is also consistent with Diaz-Seradilla et al. [17]. Karlsson et al. [15] also found a higher number of accelerations and decelerations on the day preceding and more sprint distance covered on the day succeeding the highest overall day, which we did not observe considering SpD covered, although metrics are not directly comparable. That MD contains the highest external load is also supported by previous studies [15,17,18]. Compared to studies on male players, however, our results are similar to Akenhead et al. [38] and Anderson et al. [39], who examined the external training load of English Premier League teams. Together, they both show a pattern of higher loads preceding and succeeding days of lower loads irrespective of metric, similar to our study. In addition, in a study on a team from the Eredivisie, Stevens et al. [40] noted that relative to match values (100%), accelerations and decelerations (39–90%) were much higher compared to the other metrics, which mirrors our study (27–79%).

A major strength of this study is that we utilized a multi-team, multi-season approach, in contrast to most other observational studies in football, which usually are one-team, one-season. We also examined external training load across a range of cycle lengths with different numbers of days between matches. This complements Karlsson et al. [15], who concatenated similar days in cycles with five, six, and seven days between matches while adding to Diaz-Seradilla et al. [17] and Romero-Moraleda et al. [18], who studied cycles with four and six days available, respectively. Another strength is that we compared external training load for both starters and non-starters, which, as far as we know, has not been investigated in women’s elite football.

There are also some limitations to our approach. Mainly, we lacked context surrounding each training day, thus making it very hard to discern whether training had occurred or not on observations with missing data. Therefore, some observations were likely imputed when they should have been removed from the dataset. Also, the categories of starters and non-starters could be viewed as somewhat crude. For example, we put starters who were subbed out early and those who played the whole game within the starter category. However, if we were to dichotomize further, this would only run into the problem of where to set the cut-off point, and thus, we thought it was better to leave this category as it was.

Our study serves as a springboard for future research endeavors to refine our understanding of external training load dynamics in professional football. To enhance precision, future studies should gather contextual information surrounding each training day, including details on specific drills and focus areas. Refining player categories to capture more nuanced distinctions, such as players substituted early versus those playing the whole game, can provide additional insights into training load variations. In addition, longitudinal studies spanning multiple seasons and teams can reveal evolving trends influenced by changes in coaching staff, coaching strategies, or other external factors. Finally, investigating the impact of different training formats, including small-sided games and specific drills, on external training load can guide coaches in designing more effective training sessions.

### Practical applications

The insights gained from our study have several practical implications for the training and management of professional football players. First, our study identifies a sound approach to training load periodization, wherein the emphasis of a high load day, succeeding and preceding days of lower load, is well within the recommendations of contemporary training theory. Practitioners can thus leverage the findings of our study as a template or starting point when designing their training programs. Furthermore, our observation of a possible disparity between the players’ maximum achievable speed and their training speeds highlights an area for improvement. Combined with the fact that the volume of sprinting is comparably low especially regarding acceleration, this could point to a neglect of speed work in the daily training regimen of female football players. Thus, coaches should be mindful of incorporating exercises that allow players to reach top speeds. This not only enhances speed-specific capacities but may also contribute to injury prevention. Finally, our findings raise awareness of potential load compensation for non-starters, prompting coaches to explore compensatory strategies for optimal player development.

## Conclusion

These results provide further evidence regarding how highly trained female football teams adjust their external training load across various microcycles. Loading patterns typically take on a shape similar to a pyramid, or a skewed pyramid, during longer cycle lengths, with higher training loads towards the middle compared to the start and the end of the cycle. Non-starters reached higher peak speeds and covered more total distance and combined acceleration- and deceleration distance on MD+2 in cycles with 3–6 days between matches. However, there was no significant load differentiation from MD-5 and onwards.

## Supporting information

S1 Fig External training load by number of days between matches and in proximity to match day (non-imputed data). (TIFF)

S1 Table Between-day contrasts from imputed data. (XLSX)

S2 Table Estimated marginal means by MD, cycle, and squad status, from imputed data. (XLSX)

S3 Table Starter vs. non-starter contrasts from imputed data. (XLSX)

S4 Table Between-day contrasts from non-imputed data. (XLSX)

S5 Table Estimated marginal means by MD, cycle, and squad status, from non-imputed data. (XLSX)

S6 Table Starter vs. non-starter contrasts from non-imputed data. (XLSX)
