# Supplementary figures and images for: An analysis of training load in highly trained female football players
Source: PLoS One. 2024 Mar 28;19(3):e0299851. doi: 10.1371/journal.pone.0299851 (PMC10977714; doi:10.1371/journal.pone.0299851)

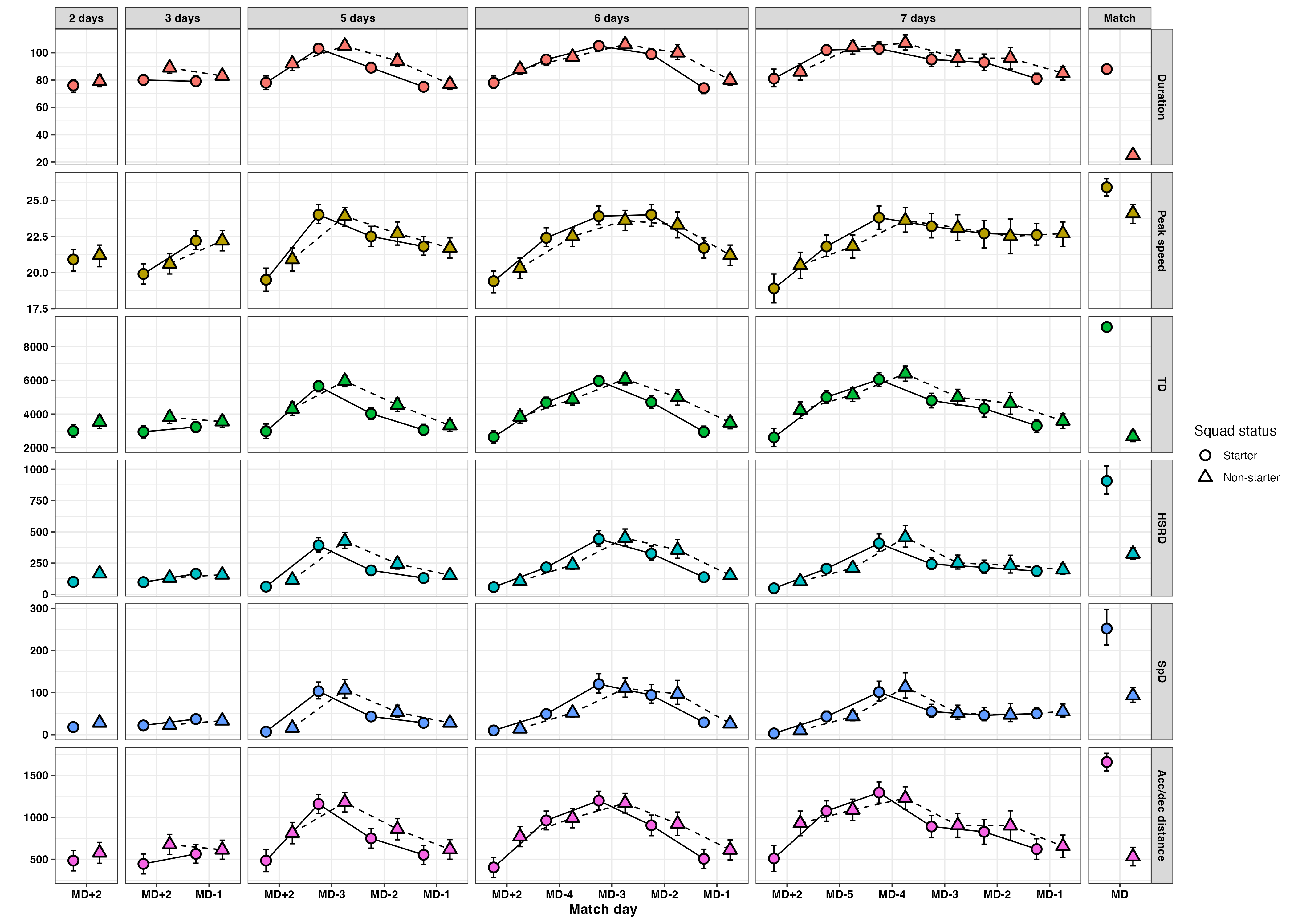

Supplement: S1 Fig — (TIFF) [file pone.0299851.s001.tiff]
